# Local Antimicrobial Potential of Bupivacaine and Tolfenamic Acid-Loaded Ultra-High Molecular Weight Polyethylene (UHMWPE) for Orthopedic Infection

**DOI:** 10.3390/bioengineering12020173

**Published:** 2025-02-12

**Authors:** Amita Sekar, Nicoletta Inverardi, Sashank Lekkala, Andrew Thomson, Vikram Daesety, Darina Trendafilova, Peyton Tierney, Jamie E. Collins, Orhun K. Muratoglu, Ebru Oral

**Affiliations:** 1 Harris Orthopaedics Laboratory, Massachusetts General Hospital, Boston, MA 02114, USA; asekar2@mgh.harvard.edu (A.S.);; 2 Department of Orthopaedic Surgery, Harvard Medical School, Boston, MA 02115, USA; 3 Department of Orthopaedic Surgery, Brigham and Women’s Hospital, Boston, MA 02115, USA

**Keywords:** orthopedic infections, implant materials, ultra-high molecular weight polyethylene, UHMWPE, local drug delivery, repurposed drugs, bupivacaine hydrochloride, tolfenamic acid, antibacterial activity, *Staphylococcus aureus*, prophylaxis

## Abstract

Peri-prosthetic joint infection (PJI) is a major post-arthroplasty complication that warrants alternative antibacterial approaches to improve prophylaxis and treatment outcomes. Local administration of analgesics post-surgery is common. Recent studies have demonstrated the antimicrobial potential of analgesics and the feasibility of dual drug-eluting ultra-high molecular weight polyethylene (UHMWPE) for local antibacterial applications. However, the antibacterial mechanism of action is poorly understood, and the translational value of antimicrobial dual drug-loaded UHMWPE has not been evaluated. In this study, we utilized the Laurdan assay and gene expression analysis to demonstrate the antibacterial action of bupivacaine hydrochloride (BP) and tolfenamic acid (TA) against *Staphylococcus aureus.* Furthermore, we incorporated BP and TA into UHMWPE at different weight concentrations and studied their longitudinal drug release and real-time antibacterial properties. The analgesics showed a significant effect on the bacterial membrane properties comparable to known antibiotics and regulated bacterial gene expression. For the dual drug-loaded UHMWPE, the drug release rate from BP/TA combinations was interestingly not a direct function of the loaded drug weight percent, potentially due to the hydrophobicity of TA and the interactions between the two drugs. Combinations of BP and TA at the higher total drug concentration (10 and 20%) showed a prolonged antibacterial effect against *S. aureus*, with great potential for prophylactic use.

## 1. Introduction

Peri-prosthetic joint infection (PJI) has been recognized as a devastating complication associated with total joint arthroplasty (TJA) with high morbidity and mortality rates [1,2,3]. Two-stage revision surgery remains the standard procedure for managing PJI, which involves tissue debridement, the removal of infected implant components, prolonged antibiotic therapy, and replacement with new implant materials [4,5,6]. The primary causative organism is *Staphylococcus aureus*, which adheres to the implant materials following surgical site contamination and colonizes the joint space, eventually resulting in deep bone infections [7,8]. There is a high revision failure rate associated with PJI incidence due to the presence of *S. aureus* biofilms, which interferes with the effective eradication of PJI [9,10,11]. It has been reported that biofilms provide a physical and chemical protective barrier against host responses and antibiotic treatment [12]. Furthermore, biofilm formation promotes antibiotic tolerance and an increase in the protected transfer of antibiotic resistance [13,14,15]. This is the main reason why antibiotics can fail to eradicate biofilms, despite being the most effective treatment against it.

Currently, treatment approaches for PJI heavily rely on the systemic dosing of a cocktail of antibiotics for a prolonged period. Common empirical therapy involves broad spectrum β-lactam or fourth-generation cephalosporins along with vancomycin, and adjunctive agents such as rifampicin are used when needed [16,17]. For prophylaxis, antibiotic-loaded bone cement is commonly used for the local delivery of aminoglycosides such as gentamicin [18]. Clinical breakpoints derived from the antibiotic susceptibility profiles of bacteria in suspension are typically used to guide dosing decisions. However, in PJI, the risk and severity associated with implant-adhered *S. aureus* biofilms often warrant the prolonged application of high doses of antibiotics (>100× MIC) locally to ensure the efficient eradication of infection and to improve treatment outcomes [19]. Drug delivery devices that can locally release therapeutics to increase the bioavailability and efficacy of the drugs can be an additional tool for the treatment of PJI [18]. Ultra-high molecular weight polyethylene (UHMWPE) is routinely used as the preferred bearing component in total joint replacement implants, and a drug delivery device made from UHMWPE could provide not only load-bearing and function, but also the sustained release of high concentrations of the drug. Antibiotic-loaded UHMWPE implant materials have been developed to address this gap [20,21,22].

While antibiotics remain our strongest tool against infections, antibiotic resistance poses a significant global health threat, with over 2.8 million cases reported annually in the U.S. [23]. The National Action Plan outlines strategic steps to address this issue including slowing the emergence of antibiotic resistance, preventing the spread of resistant infections, and accelerating research on new treatments. In PJI, biofilm-associated antibiotic tolerance is a particularly prominent problem due to the presence of avascular orthopedic hardware [24,25]. Therefore, it has become necessary to seek alternative strategies to bolster the efficacy of antibiotics in the face of an overall increase in PJI risk and severity.

One important strategy is the repurposing of drugs commonly used for other indications. In our previous work, we showed that the analgesic bupivacaine (BP) and the non-steroidal anti-inflammatory drugs (NSAID) tolfenamic acid (TA) and ketorolac in their free drug form [26,27] possessed antimicrobial activity by themselves and could act synergistically with gentamicin and vancomycin against *S. aureus*. Detailed studies on repurposed drugs have demonstrated mechanisms of action against bacteria through cell wall/membrane disruption, metabolic pathway inhibition, and DNA damage, comparable to known antibiotic action [28,29,30]. However, there is limited information regarding the exact mechanism of the antibacterial action of pain medications. Analgesic-loaded or doped UHMWPE can be an additional tool contributing to multi-modal prophylaxis and the treatment of post-arthroplasty associated infection besides providing pain management [31,32]. In this context, we have previously demonstrated the synergistic antibacterial activity of BP and TA and determined an optimal range for the loading ratio of these two drugs in UHMWPE for surface protection [33]. Here, we investigated the effect of total drug dosing as well as the longitudinal antimicrobial effect of the eluted drugs. In this study, we also aimed to understand the antibacterial activity of BP and TA by studying its effect on the bacterial membrane and gene expression. The longitudinal antibacterial activity correlating to its dual drug elution kinetics were evaluated using a purposely designed methodology based on real-time microbial viability detection. This study will further our understanding of the antimicrobial potential of analgesics and the translational value of dual analgesic-loaded UHMWPE for PJI prophylaxis and treatment.

## 2. Materials and Methods

### 2.1. Bacteria, Drug Stock, and Material Preparation

*S. aureus* ATCC 12600 was used in this study. The frozen stocks of bacterial culture were thawed and grown overnight (18–24 h) in tryptic soy agar plates (TSA) at 35 °C. The *S. aureus* colonies were cultured in tryptic soy broth (TSB) or agar (TSA) at 35 °C overnight (18–24 h). The turbidity of the broth cultures was measured at 600 nm and adjusted to ~10^5^ CFU/mL using an enumeration curve or real-time microbial viability assay (BacTiter Glo^®^, Promega, Madison, WI, USA). BP HCl (PHR1128, Sigma Aldrich, St. Louis, MO, USA) and TA (70480, Cayman Chemical, Ann Arbor, MI, USA) were purchased as crystalline solid powders. BP was solubilized in deionized water (solubility < 50 mg/mL) and TA was dissolved in DMSO for drug stock preparation, then diluted in deionized water by keeping the volume concentration of DMSO < 5% while performing antibacterial activity assays.

Ultra-high molecular weight polyethylene (medical grade UHMWPE, GUR^®^ 1020) (Celanese Corporation, Irving, TX, USA) was used. The drug powder was sieved through a 75 µm mesh. Blending was performed by the mechanical mixing of the UHMWPE powder (mean particle size of 150 µm) with the sieved drug powder (powder fraction < 75 µm) for 30 min through a Turbula^®^ shaker (Willy. A. Bachofen AG, Muttenz, Switzerland). Blends were prepared at a total drug loading of 10 or 20 wt. % as single drug-loaded compositions or as dual drug-loaded compositions with both BP and TA, according to the formulations listed in Table 1. Virgin UHMWPE (i.e., without drug loading) was used as the control. The formulations were processed by compression molding (Carver Press, Carver, Wabash, IN, USA) by filling a custom mold (cavity: 50 × 85 mm^2^) with the blend and applying a compressive force at 170 °C for 10 min (pressure: 20 MPa) followed by cooling under pressure to room temperature for at least 45 min. Blocks with a thickness of about 4 mm were obtained and machined by a benchtop CNC machine (ShopBot Desktop, ShopBot Tools, Inc., Durham, NC, USA) into the prismatic samples (3 × 5 × 20 or 3 × 5 × 10 mm^3^) used for testing.

### 2.2. Antibacterial Activity of Analgesics

The MIC of BP and TA against *S. aureus* was determined according to the established broth microdilution protocol (CLSI M07-A10). Briefly, drug stocks were serially diluted in Mueller Hinton Broth (MHB) and ~10^5^ CFU was incubated with the drug concentrations for a period of 18–24 h at 35 °C. Bacteria with no drug exposure and blank media served as internal controls for the experiment. The MIC was determined as the minimum concentration of respective drugs that inhibited the growth of bacteria as indicated by clear wells. The MBC was determined by plating samples on TSA from the MIC and three more wells that showed no turbidity.

### 2.3. Bacterial Membrane Fluidity Analysis

The Laurdan assay was performed on *S. aureus* to determine the effect of BP and TA on the bacterial membrane fluidity dynamics. Briefly, overnight, *S. aureus* cultures were diluted and adjusted to 10^8^ CFU/mL, and the bacteria were centrifuged at 6000× *g* for 5 min and washed 3× with sterile 1 × phosphate buffered saline (PBS). The bacteria were then stained for 10 min with 10 µM Laurdan reagent (Invitrogen™ Carlsbad, CA, USA), and using centrifugation, the excess stain on the bacteria was washed using the Laurdan assay buffer (5×). The stained bacteria were then exposed to MIC and sub-MIC (0.5× MIC, 0.25× MIC) concentrations of BP and TA, and the fluorescence units (λ_ex_ = 350 nm, λ_em_ = 435, 500 nm) from bacterial membrane-bound Laurdan was measured every 10 min for a period of 40 min. Benzyl alcohol-treated bacteria served as the positive control to measure fluidization of the bacterial membrane. Bacteria with no drug exposure served as the internal control. The generalized polarization of membrane-bound Laurdan fluorescence was calculated using the following equation: Laurdan GP = (i435 nm − i500 nm)/(i435 nm + i500 nm), where *i* is the intensity of fluorescence [34].

### 2.4. Gene Expression Studies

Overnight cultures of *S. aureus* adjusted to 10^8^ CFU/mL were exposed to inhibitory and sub-inhibitory concentrations (MIC, 0.5× and 0.25× MIC) of BP and TA for a period of 1 h. Bacteria with no drug exposure served as the internal control. The treated and non-treated bacteria were pelleted by centrifugation at 6000× *g*, 5 min, and were washed with ice-cold PBS (3×). The bacteria pellet was then subjected to lysostaphin (100 µg/mL) treatment at 37 °C for 10 min and underwent mechanical lysis using acid-washed beads. The lysate was used to extract RNA using an RNeasy kit according to the manufacturer’s protocol (Qiagen, Hilden, Germany). The RNA concentration was spectrophotometrically determined using NanoDrop One (ThermoScientific, Waltham, MA, USA). Bacterial gene expression was performed for *S. aureus vraR*, *ebpS*, and *icaA* using specific primers (Table 2). *S. aureus 16srRNA* served as a housekeeping gene to perform relative gene expression analysis using the 2^(−ΔΔCq)^ method.

### 2.5. Drug Release Profile of BP/TA-Loaded UHMWPE

Drug release studies were performed by eluting samples (3 × 5 × 20 mm^3^, *n* = 6) into deionized water (1.7 mL) at room temperature under mild shaking. Eluent collection was performed at given timepoints (6 h, 1, 2, 3 days, 1, 2, and 3 weeks), and fresh medium was replenished at each point. The concentration of the drugs in the eluent was determined spectrophotometrically by measuring their UV absorbance with a plate reader (BioTek, Synergy H1, Agilent, Santa Clara, CA, USA). The wavelength used for measuring BP was 250 nm, and the one for measuring TA was 285 nm. The drug mass released was used to build cumulative drug mass and fractional release curves, and the release rate profiles as a function of the elution time.

### 2.6. Longitudinal Antibacterial Activity of BP/TA-Loaded UHMWPE

The antibacterial activity of the analgesic-loaded UHMWPE samples was tested using a purposely developed semi-static method based on a chemiluminescent assay [35]. Using a 10 mL syringe setup, six 3 × 5 × 10 mm^3^ strips were incubated with 10^5^ CFU/mL *S. aureus* suspension in Mueller Hinton Broth (3.1 mL) at 37 °C, 100 rpm. At each timepoint (i.e., 6 h, 1, 2, 3, and 7 days), 0.5 mL of the bacterial suspension was collected. A total of 100 μL of suspension was mixed with 100 μL of the BacTiter-Glo™ reagent and its luminescence was measured using the microplate reader. The observed luminescence values were correlated to bacteria viability (CFU/mL) using a calibration curve (y = 0.1075x^2^ − 0.5512x + 2.8533; detection limit ~10^3^ CFU/mL). Centrifugation was performed on the remaining solution at 10,000× *g*, 10 min for pelleting the bacterial population. The spent media were discarded, and pellet was resuspended in fresh MHB. Then, 10 µL of the bacterial suspension was also plated on TSA to verify the bacteria viability. Virgin UHMWPE was used as a positive control. The mean concentration of the drug in bacterial broth was estimated by scaling the released drug for the surface area of the sample and considering the mean for each time period (i.e., 0–6 h, 6 h–1 d, 2–3 d, 3–7 d).

### 2.7. Statistics

The longitudinal antibacterial activity data of the BP/TA-loaded UHMWPE was analyzed using the 2-way ANOVA test and the Benjamini–Hochberg method to adjust for multiple testing. The Student t-test was performed on the relative gene expression data. *p*-values < 0.05 were considered statistically significant.

## 3. Results

### 3.1. Antibacterial Properties of BP and TA Against S. aureus

**Figure 1:**
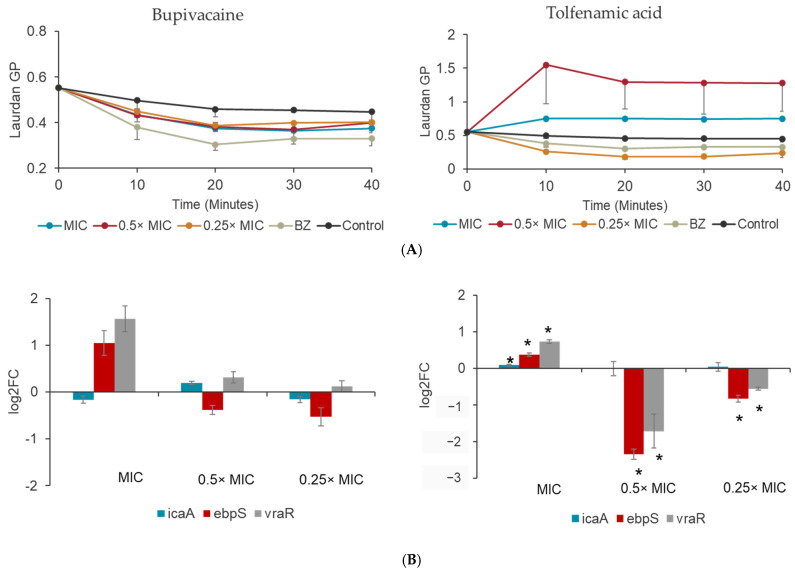
(**A**) Membrane fluidity analysis of *S. aureus* exposed to inhibitory and sub-inhibitory concentrations of BP and TA using the Laurdan reagent. The Laurdan GP of benzyl alcohol (BZ) served as the positive control. (**B**) Relative gene expression analysis of the *icaA*, *vraR*, and *ebpS* genes of *S. aureus* exposed to MIC, 0.5× MIC, and 0.25× MIC of BP and TA. * indicates *p* < 0.05 vs. control.

The minimum inhibitory concentration of BP and TA against *S. aureus* ATCC 12600 was 1.75 ± 0.75 mg/mL and 62.5 ± 0 µg/mL, respectively. The *S. aureus* membrane dynamics were assessed to evaluate the potential of analgesics to trigger membrane fluidity or rigidity. The inhibitory and sub-inhibitory concentrations (0.5× MIC and 0.25× MIC) showed decreased Laurdan GP values for the *S. aureus* membrane within 10 min of exposure compared with the no drug control (Figure 1A). The GP values of BP-exposed *S. aureus* were comparable to the bacteria exposed to the membrane fluidizing agent benzyl alcohol that served as the positive control for the experiment. The inhibitory and sub-inhibitory concentrations of TA (MIC, and 0.5× MIC) triggered an increase in the Laurdan GP values for *S. aureus* within 10 min of exposure, with the 0.5× MIC concentration exhibiting a significant increase in the Laurdan GP values compared with the inhibitory concentrations of TA. The 0.25× MIC GP values were comparable to those of the control bacteria without drug exposure (Figure 1A).

Gene expression studies were performed on the BP- and TA-treated *S. aureus* to determine the expression changes in the bacterial genes *vraR*, *icaA*, and *ebpS*. In bacteria exposed to inhibitory concentrations of BP, the gene expression of *icaA* remained unaltered compared with the control bacteria without drug exposure. The *ebpS* and *vraR* gene expression were significantly elevated (>1 log) compared with the control. On the contrary, the sub-inhibitory concentration of BP did not trigger drastic fold change differences for *vraR*, *icaA*, and *ebpS* compared with the control, even though the observed changes were significant. In bacteria exposed to inhibitory concentrations of TA, all of the genes were somewhat regulated (<1 log). The sub-inhibitory concentration of TA, 0.5× MIC, significantly downregulated *ebpS* and *vraR* (>1.5 log), and 0.25× MIC significantly downregulated *ebpS* and *vraR* (>0.5 log) compared with the control bacteria without drug exposure. The *icaA* expression remained unaltered in the presence of sub-inhibitory concentrations of TA (Figure 1B).

### 3.2. Longitudinal Assessment of Drug Release and Antibacterial Activity

**Figure 2:**
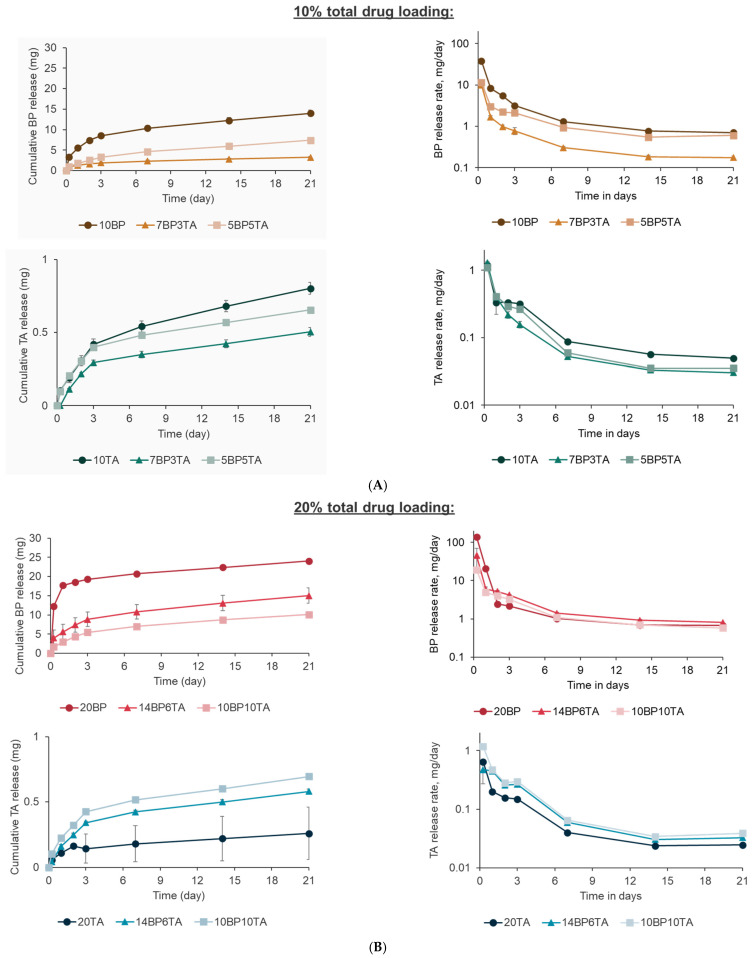
Cumulative drug release of BP and TA and the drug release rate (determined for a hypothetical 10 cm^2^ implant surface area) for the (**A**) 10% total drug-loaded UHMWPE for 21 days and (**B**) 20% total drug-loaded UHMWPE for 21 days.

The elution of BP and TA out of the polymeric matrix was very different. More BP could be released compared with TA over the course of the experiment (21 days) for all loading conditions (Figure 2A,B). There was generally an increasing trend in cumulative BP release as the drug loading of BP increased (maximum of ~24 mg at day 21 for 20BP loaded UHMWPE). The burst release rates (6 h) for UHMWPEs with a total drug loading of 20% (maximum of ~140 mg/day) were higher than those with a total drug loading of 10% (maximum of ~40 mg/day). The release rate of BP remained higher than 2 mg/day for about 3 days for the UHMWPEs with the highest drug loading (20% total drug, Figure 2B). The released amount of TA was small (maximum of 0.8 mg at day 21) and was not dependent on drug loading (Figure 2B). The release rates of TA remained significantly higher than 0.15 mg/day for 3 days for all UHMWPEs (Figure 2A,B).

**Figure 3:**
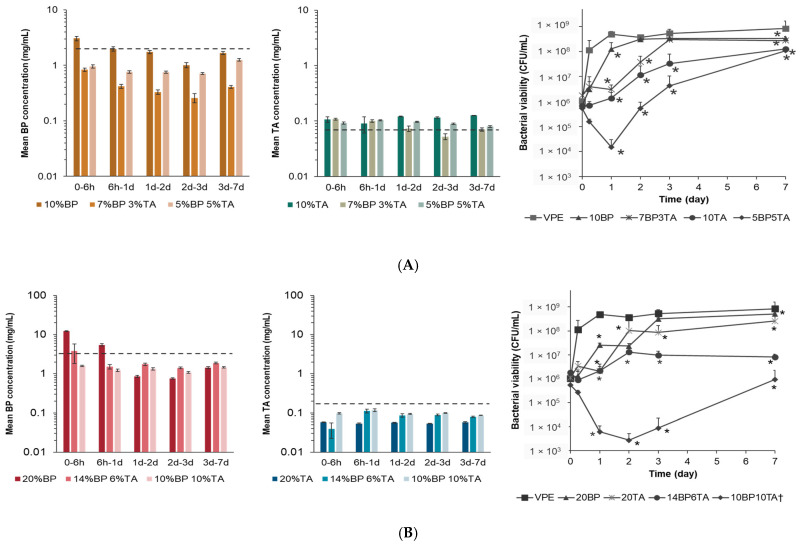
(**A**) Concentration of BP and TA released from the 10% *w*/*w* drug-loaded strips (3 × 5 × 10 mm^3^) and their antibacterial activity was assessed by the BacTiter-Glo assay. (**B**) Concentration of BP and TA released from the 20% *w*/*w* drug-loaded strips (3 × 5 × 10 mm^3^) and their antibacterial activity assessed by the BacTiter-Glo assay. * indicates *p* < 0.05 vs. VPE. *p*-values were determined from the 2-way ANOVA adjusted with the Benjamini–Hochberg procedure. The minimum inhibitory concentration (MIC) for *S. aureus* is shown as a dotted line.

The UHMWPE formulations were incubated with a 10^5^ CFU/mL bacterial inoculum. The bacterial viability was measured as the drug eluted out of the polymeric matrix using a semi-static protocol. The 10BP-loaded UHMWPE could inhibit bacterial growth only at the first timepoint, as the eluted BP concentration (~2 mg/mL) was greater than the MIC. As the eluted concentration fell below the MIC after day 1, the bacterial growth increased to 10^8^ CFU/mL, similar to VPE (Figure 3A). The 10TA-loaded UHMWPE released concentrations above the MIC for the duration of the study. Consequently, the bacterial viability was inhibited for the first few timepoints; thereafter, bacterial growth up to 10^8^ CFU/mL occurred on day 7. Dual drug-loaded UHMWPE with a total drug loading of 10 wt% showed an initial inhibition in bacterial growth, and the composition with the highest percentage of TA (5BP5TA) could inhibit bacterial growth up to day 3 when compared with 7BP3TA. For the 5BP5TA composition, not only was the released TA concentration higher than the MIC for the whole duration, but the released BP concentration was also significantly higher than the one released by the 7BP3TA composition (Figure 3A).

Among the compositions with 20 wt% total drug loading, 20BP and 20TA demonstrated an initial inhibition of the bacterial growth for only the first 6 h burst release for BP and up to day 1 for TA, respectively. The released concentrations were above the MIC up to day 1 for BP and consistently below the MIC for TA. The 14BP6TA sample could inhibit bacterial growth until day 7, whereas the TA concentration released was lower than the MIC at 6 h but increased above the MIC thereafter. The 10TA10BP sample had the best antibacterial performance as it could significantly decrease bacterial viability close to the detection limit until day 3. For the 10BP10TA composition, the TA concentration at 6 h was above the MIC (Figure 3B).

## 4. Discussion

PJI remains one of the most challenging complications associated with orthopedic implants. Bacterial biofilm formation on the implant and surrounding joint tissue and its associated antibiotic tolerance and resistance pose a significant problem that warrants effective and novel prophylactic and treatment approaches [24,36,37]. Previously, we have demonstrated the growth dynamics and evolution of antibiotic tolerance and resistance for implant-associated *S. aureus* infections in vivo [19,27]. To circumvent the challenges associated with antibiotics, synergistic formulations and repurposed drugs could be promising tools that could aid in developing preventive and therapeutic orthopedic drug delivery applications, which is our long-term goal. In this study, we assessed the potential of two drugs, BP and TA, for their antibacterial properties and evaluated the translational efficacy of high percentage (*w*/*w*) dual-analgesic loaded UHMWPE by correlating the release kinetics and their antibacterial activity.

The observed minimum inhibitory concentrations (MICs) of BP (1.75 ± 0.75 mg/mL) and TA (62.5 ± 0 µg/mL) against *S. aureus* ATCC 12600 highlight their potential to act as antimicrobial agents. The key feature of antibiotics is their mechanism of action, which ranges from interactions with the bacterial cell wall (i.e., penicillin, vancomycin, and daptomycin) to affecting bacterial efflux pumps and biosynthesis processes (gentamicin) [38,39,40]. Repurposed drugs have shown significant promise, as approximately 30% of the U.S. FDA-approved drugs are repurposed, enabling their expanded use [41]. High-throughput screening utilizing systems biology and integrative multi-omics approaches are the norm for the drug discovery of non-antibiotics with potential antimicrobial activity [42]. Detailed studies have revealed specific mechanisms of action for repurposed drugs against Gram-positive and Gram-negative bacteria. Drugs such as halicin and fluoxetine have been shown to disrupt the bacterial membrane, effectively targeting multi-drug resistance [43,44]. The mechanisms of action for BP (sodium ion channel blocker) and TA (prostaglandin biosynthesis inhibitor) were similarly explored in this study for their impact on bacterial membrane dynamics using Laurdan generalized polarization (GP) values. BP exposure led to a significant decrease in GP values, suggesting increased membrane fluidity similar to antibiotics such as vancomycin and gentamicin [44]. The result was comparable to benzyl alcohol, a known membrane fluidizing agent, indicating that BP destabilizes the bacterial membrane. Such disruption can compromise the bacterial membrane’s integrity, leading to cell death. Conversely, TA exhibited the opposite effect by increasing the GP values at inhibitory and sub-inhibitory concentrations (0.5× MIC), suggesting membrane rigidification comparable to antibiotics such as daptomycin [40]. Interestingly, the 0.5× MIC concentration had a more pronounced effect on increasing the GP values compared with the MIC, indicating concentration-dependent interactions with the bacterial membrane. At 0.25× MIC, TA’s effect was minimal, resembling the control. These findings imply that BP and TA employ distinct mechanisms of membrane fluidization and rigidification, respectively, that contribute to their antibacterial activity.

Antimicrobial activity is closely tied to the bacterial response to the drug action, which can be determined using gene expression studies. The presence of antibiotics triggers critical cell wall biosynthesis, ion transport, and nucleic acid metabolic processes that determine the outcomes of the antibacterial efficacy of the drugs [45,46,47]. The adverse drug action can also unexpectedly promote bacterial survival and biofilm formation in the case of gentamicin [48]. In this study, the expression profiles of the cell-wall biosynthesis associated *vraR*, bacterial adhesion-associated *ebpS*, and biofilm formation-associated *icaA* were determined in the presence of BP and TA to provide further insights into their effects on bacterial stress and virulence pathways. BP at inhibitory concentrations significantly upregulated (>1 log) genes associated with the bacterial stress response (*vraR*) and extracellular matrix proteins (*ebpS*), respectively. On the contrary, sub-inhibitory concentrations of BP did not induce drastic changes in gene expression. The upregulation of *vraR* and *ebpS* in the presence of inhibitory concentrations of BP suggests an attempt by *S. aureus* to respond to membrane stress induced by BP and promote adhesion. In contrast, TA had a concentration-dependent regulatory effect. At sub-inhibitory concentrations (0.5× and 0.25× MIC), *vraR* and *ebpS* were significantly downregulated (>1.5 log and >0.5 log, respectively). The downregulation of these genes at sub-inhibitory concentrations suggests a unique property of TA to interfere with stress-response pathways, potentially reducing bacterial resistance mechanisms and impairing biofilm formation. The expression of *icaA*, a key gene for polysaccharide intercellular adhesin production and biofilm formation, remained unaltered under both BP and TA exposure, suggesting that the drugs’ primary mode of action does not directly target biofilm production at the genetic level.

UHMWPE loading with analgesics and NSAID was already demonstrated in our previous work, where we showed that BP and TA could be loaded into UHMWPE, and their elution was controlled by their morphology [31]. BP and TA are moderately thermally stable and their stability after high-temperature processing with UHMWPE has been previously demonstrated by NMR [32,49,50]. These blends could also be sterilized by radiation exposure without affecting the stability of the drugs. This was particularly important for obtaining a crosslinked network to achieve suitable wear properties. Thus, the translational value of these blends is promising, and furthering the understanding of their antibacterial properties is of high significance. The difference in the physical properties of BP HCl (hydrophilic and polar) and TA (hydrophobic and non-polar) determines their elution behavior and kinetics. The salt form of BP is hydrophilic, and it is known to lead to a phase-separated structure when blended with the hydrophobic UHMWPE. TA is highly hydrophobic and potentially partially soluble inside the hydrophobic UHMWPE matrix [34]. The longitudinal assessment of drug release from the UHMWPE matrices revealed stark differences between BP and TA, influencing their antibacterial performance. BP demonstrated higher cumulative release compared with TA, with an increasing trend correlating with drug loading (e.g., ~24 mg for 20BP at day 21). However, the BP release rates exhibited a burst release within the first 6 h, followed by a sharp decline. The highest burst release for BP was observed at 20% drug loading (~140 mg/day), but the concentrations fell below the MIC after 1 day, allowing bacterial regrowth. In contrast, TA displayed a sustained release profile with minimal dependence on drug loading, thus contributing to prolonged antibacterial activity.

For drug-delivery applications, standard antibacterial activity evaluation methods lack real-time microbial viability data corresponding to the drug-release profiles [51]. Consequently, a longitudinal analysis of the drug-delivery application to evaluate its activity potential cannot be performed. To address this gap, a semi-static method based on the elution kinetics experimental setup was purposely designed to determine the antibacterial activity kinetics and thereby its translational value [35]. The antibacterial efficacy of these formulations was evaluated under a bacterial inoculum of 10^5^ CFU/mL. The 10BP-loaded UHMWPE inhibited bacterial growth only initially (eluted concentration > MIC), but failed to sustain efficacy as the concentrations dropped below MIC after 1 day, mirroring the burst-release kinetics. In contrast, the 10TA-loaded UHMWPE exhibited antibacterial activity for the first few timepoints, after which bacterial regrowth occurred at day 7. Dual drug-loaded formulations demonstrated a superior performance, combining the benefits of both drugs. At 10% total drug loading, the 5BP5TA sample achieved extended bacterial inhibition up to day 3, outperforming the 7BP3TA composition. This result can be attributed to the sustained release of TA and higher BP concentrations at early timepoints. At 20% total drug loading, the 14BP6TA formulation inhibited bacterial growth for 7 days, likely due to a more balanced release profile of both drugs. The 10TA10BP composition exhibited the best antibacterial performance, reducing the bacterial viability close to the detection limit up to day 3. Notably, this composition maintained TA concentrations above MIC at the critical 6-h timepoint, emphasizing the importance of achieving therapeutic drug levels early to prevent bacterial colonization and its value as a prophylactic application.

The findings of this study highlight the potential of BP and TA for preventing and managing PJIs. The insights into the mechanism of the antibacterial action of BP and TA could aid in rational repurposing and synergistic use with reduced side effects [52,53]. BP’s rapid burst release and membrane fluidizing effects make it a valuable candidate for early-stage infection prevention immediately after implantation. However, its limited antibacterial activity necessitates combination strategies. TA’s sustained release, membrane-rigidifying effects, and downregulation of bacterial stress genes suggest its utility in prolonged infection prevention. Dual drug-loaded formulations, particularly 5BP5TA, 10TA10BP, and 14BP6TA, demonstrate synergistic effects by providing both immediate and sustained antibacterial activity. In addition, TA is non-inflammatory, the effects of which can be beneficial in managing post-surgical pain and complications [54]. These compositions are promising candidates for further preclinical evaluation as they address both early bacterial colonization and prolonged inhibition, two critical aspects of PJI prevention.

## 5. Conclusions

The findings underscore the importance of drug release kinetics, membrane-targeting mechanisms, and bacterial gene regulation in determining the efficacy of UHMWPE-based drug-eluting systems for PJI prevention. Revealing the mechanism of the antibacterial action of BP/TA and the subsequent longitudinal assessment of their synergistic application have furthered our understanding of the antimicrobial potential of analgesics and the translational value of dual analgesic-loaded UHMWPE for PJI prophylaxis and treatment. Dual-drug systems combining BP and TA show enhanced antibacterial performance, offering a potential solution for mitigating PJI risk associated with orthopedic implants. Furthermore, in vivo studies will be essential to validate these findings and optimize formulations for clinical applications.

## Figures and Tables

**Table 1:** List of drug-loaded UHMWPE formulations tested.

Formulation	BP HCl [% *w*/*w*]	TA [%*w*/*w*]	Code
Virgin UHMWPE	0	0	VPE
10% *w*/*w*	10	0	10BP
	7	3	7BP3TA
	5	5	5BP5TA
	0	10	10TA
20% *w*/*w*	20	0	20BP
	14	6	14BP6TA
	10	10	10BP10TA
	0	20	20TA

**Table 2:** List of primers.

*Gene*	Primer Sequences
*vraR*	FP 5′-AACTCTGCGCGCTTTTTCAT-3′
	RP 5′-ATATCGCCGATGCAGTTCGT-3′
*icaA*	FP 5′-TTGTCGACGTTGGCTACTGG-3′
	RP 5′-GCGTTGCTTCCAAAGACCTC-3′
*ebpS*	FP 5′-TACTTTGGCCATGCCACCTT-3′
	RP 5′-TGCTTCTGCCGCTTCAAAAC-3′
*16srRNA*	FP 5′-AGACCAGAAAGTCGCCTTCG-3′
	RP 5′-TCAACCGTGGAGGGTCATTG-3′

## Data Availability

All data generated or analyzed during this study are included in this published article.
